# Sintering Behavior and Environmental Safety of Ceramsites Prepared from High-Alumina Fly Ash, Coal Gangue and Waste Glass

**DOI:** 10.3390/ma19173654

**Published:** 2026-08-27

**Authors:** Hao Wang, Songhan Yang, Kaisen Yao, Lili Liu

**Affiliations:** 1 School of Energy and Environmental Engineering, University of Science & Technology Beijing, Beijing 100083, China; 2 School of Materials Science and Engineering, Peking University, Beijing 100871, China

**Keywords:** high-alumina fly ash, coal gangue, waste glass, ceramsite, liquid-phase sintering, leaching behavior

## Abstract

**Highlights:**

- Waste glass promoted early liquid-phase formation and broadened the sintering window.
- Optimal sintering at 1240 °C balanced low bulk density and high compressive strength.
- Leaching tests confirmed ultra-low release of hazardous elements, ensuring safety.
- Glass-assisted sintering effectively converts coal-based solid wastes into safe ceramsites.
- It demonstrates the high potential of these wastes as sustainable lightweight aggregates.
- This approach provides technical support for waste valorization and conserving resources.

**Abstract:**

High-alumina fly ash and coal gangue were used as the main aluminosilicate precursors, and waste glass was introduced as a fluxing additive to prepare waste-derived ceramsites. Based on composition design assisted by the CaO-SiO_2_-Al_2_O_3_-Na_2_O phase diagram, the effects of sintering temperature and holding time on phase evolution, microstructure, bulk density, compressive strength, and environmental safety were systematically investigated. The results showed that waste glass promoted early liquid-phase formation and broadened the effective sintering window. As the sintering temperature increased from 1200 to 1300 °C, the bulk density continuously decreased from 1.77 to 1.19 g/cm^3^, whereas the compressive strength first increased from 4.9 MPa to a maximum of 10.0 MPa at 1240 °C and then decreased to 2.8 MPa because of the competition between skeletal consolidation and excessive pore coarsening. The main crystalline phases of the ceramsites were anorthite and mullite. Prolonged holding mainly accelerated pore coalescence and pore-wall thinning, resulting in a continuous decrease in compressive strength. TCLP and long-term leaching tests conducted in freshwater, saline, and acidic media showed that the release of hazardous elements remained very low. These results demonstrate that glass-assisted sintering is an effective route for converting coal-based solid wastes into environmentally safe ceramsites.

## 1. Introduction

The continuous exploitation and combustion of coal resources have generated massive amounts of coal-based solid wastes, among which high-alumina fly ash and coal gangue are particularly abundant. The long-term stockpiling of these wastes not only occupies large areas of land but also poses persistent environmental risks due to the possible release of hazardous elements and alkaline components during weathering and leaching processes [1,2,3,4]. From the perspective of ceramic materials engineering, however, these wastes are also attractive secondary resources because they contain significant amounts of aluminosilicate phases, which are the fundamental constituents of many structural and functional ceramics [1,2,3]. Therefore, converting coal-based solid wastes into value-added ceramic products is regarded as an effective strategy for simultaneously reducing environmental burden and conserving natural mineral resources [2,3,4,5].

High-alumina fly ash is typically characterized by high contents of Al_2_O_3_ and SiO_2_, together with mullite-rich crystalline phases and a certain proportion of amorphous glassy components [1,2]. Coal gangue, although more heterogeneous and often associated with a relatively high loss on ignition, also contains abundant aluminosilicate minerals such as kaolinite and quartz [2,3,4]. Owing to their chemical and mineralogical similarity to conventional clayey raw materials, these coal-derived wastes have been widely considered promising feedstocks for ceramic fabrication, especially for lightweight aggregates, porous ceramics, and related sintered products [3,4,5,6]. Among these products, ceramsite is a representative porous aluminosilicate aggregate with broad potential applications in lightweight concrete, thermal insulation, filtration, adsorption, and ecological construction materials [5,6]. Its key properties, such as bulk density, water absorption, and mechanical strength, are closely governed by the synergistic evolution of phase composition, liquid-phase-assisted sintering, and pore structure during firing [5,6,7,8,9].

For waste-derived ceramsites, the control of liquid-phase formation is a central issue because it determines not only densification and particle bonding, but also gas entrapment, pore growth, and pore-wall stability [7,8,9,10,11]. In aluminosilicate systems, fluxing oxides such as Na_2_O, CaO, MgO, and Fe_2_O_3_ can lower the eutectic temperature, promote the formation of a liquid phase, and regulate melt viscosity, thereby strongly affecting sintering behavior and the final properties of ceramsites [7,9,11]. Recent studies have shown that the type and amount of flux components significantly influence apparent density, compressive strength, water absorption, mineral assemblage, and heavy-metal immobilization in waste-based lightweight aggregates [7,8,9,10,11]. In particular, waste glass has attracted increasing attention as a low-cost and effective fluxing additive because it is rich in alkali-bearing silicate phases and can facilitate early liquid-phase formation during firing [8,10,12]. In addition to reducing the sintering temperature, waste glass may alter the content and viscosity of the liquid phase, thereby influencing the competition between matrix densification and pore expansion. However, in systems with high proportions of coal-based wastes, the interactions among waste-glass-derived melts, aluminosilicate crystalline phases, and impurity elements remain insufficiently understood, especially with respect to phase evolution, pore-structure stabilization, and strength development [7,8,9,10,12,13].

In recent years, considerable progress has been made in the preparation of ceramsites from various solid wastes, including fly ash, sludge, tailings, steel slag, and multi-source hazardous wastes [4,6,8,9,10]. These studies have confirmed that raw-material composition, flux regulation, and firing conditions are the key factors governing the quality and performance of the final product [6,7,8,9,10]. Nevertheless, most previous studies have focused on either optimizing the raw-material ratio or evaluating the effects of single process variables on macroscopic properties. Comparatively less attention has been paid to the specific role of waste glass in high-alumina fly ash–coal gangue systems, particularly regarding how glass-assisted liquid-phase sintering regulates phase transformation, pore evolution, and the resulting balance between low density and sufficient mechanical integrity [8,9,10]. For high-alumina fly ash and coal gangue, this issue is especially important because the high Al–Si framework tends to provide structural stability, whereas the introduction of an alkali-rich glass phase may simultaneously promote sintering and intensify pore coalescence if the firing window is not properly controlled [7,8,10,13].

Beyond structural performance, environmental safety is another prerequisite for the practical application of waste-derived ceramsites. Coal-based solid wastes commonly contain trace amounts of potentially hazardous elements such as Pb, Cr, Cd, Zn, and As, and their migration behavior after high-temperature treatment must be carefully evaluated [11,14,15,16]. Previous studies have indicated that these elements can be effectively immobilized during sintering through crystal incorporation, glassy encapsulation, or the formation of relatively stable interfacial structures [14,15,16]. However, environmental assessment in many previous studies has mainly relied on short-term leaching tests, whereas long-term release behavior under different service environments has been less systematically addressed [17,18]. The toxicity characteristic leaching procedure (TCLP, U.S. EPA Method 1311) is widely used to evaluate short-term leaching risk under acidic extraction conditions, with the regulatory limits specified in 40 CFR 261.24 commonly adopted as reference criteria [19,20]. In contrast, diffusion-controlled leaching tests such as the NEN 7375 tank test can provide further information on long-term release behavior from monolithic ceramic materials under prolonged exposure [17,18,21,22]. Therefore, combining short-term TCLP evaluation with long-term multi-environment leaching tests is necessary for a more comprehensive assessment of the environmental compatibility of waste-derived ceramsites.

In view of the above considerations, this study employed a glass-assisted sintering strategy to prepare ceramsites with a high content of coal-based solid wastes using high-alumina fly ash and coal gangue as the main aluminosilicate precursors and waste glass as the fluxing additive. The effects of sintering temperature and holding time on phase composition, microstructure, bulk density, and compressive strength were systematically investigated. Meanwhile, the migration and leaching behavior of impurity elements were evaluated by both TCLP and long-term leaching tests in freshwater, saline, and acidic media. The objective of this work is to clarify how waste glass regulates liquid-phase-assisted sintering and the associated processing–structure–property relationship in high-alumina fly ash–coal gangue ceramsites, while simultaneously assessing the environmental safety of the final products. The results are expected to provide both mechanistic insight and technical support for the high-value utilization of coal-based solid wastes in sustainable ceramic materials.

## 2. Materials and Methods

### 2.1. Raw Materials and Characterization

High-alumina fly ash and coal gangue were collected from long-term stockpiles in Shuozhou, China. Waste glass, mainly composed of sodium-silicate-based glass, was used as a fluxing additive. All raw materials were first dried at 105 °C for 24 h to constant mass, crushed, and sieved to pass through a 200-mesh sieve prior to use. No further chemical pretreatment was applied.

**Figure 1:**
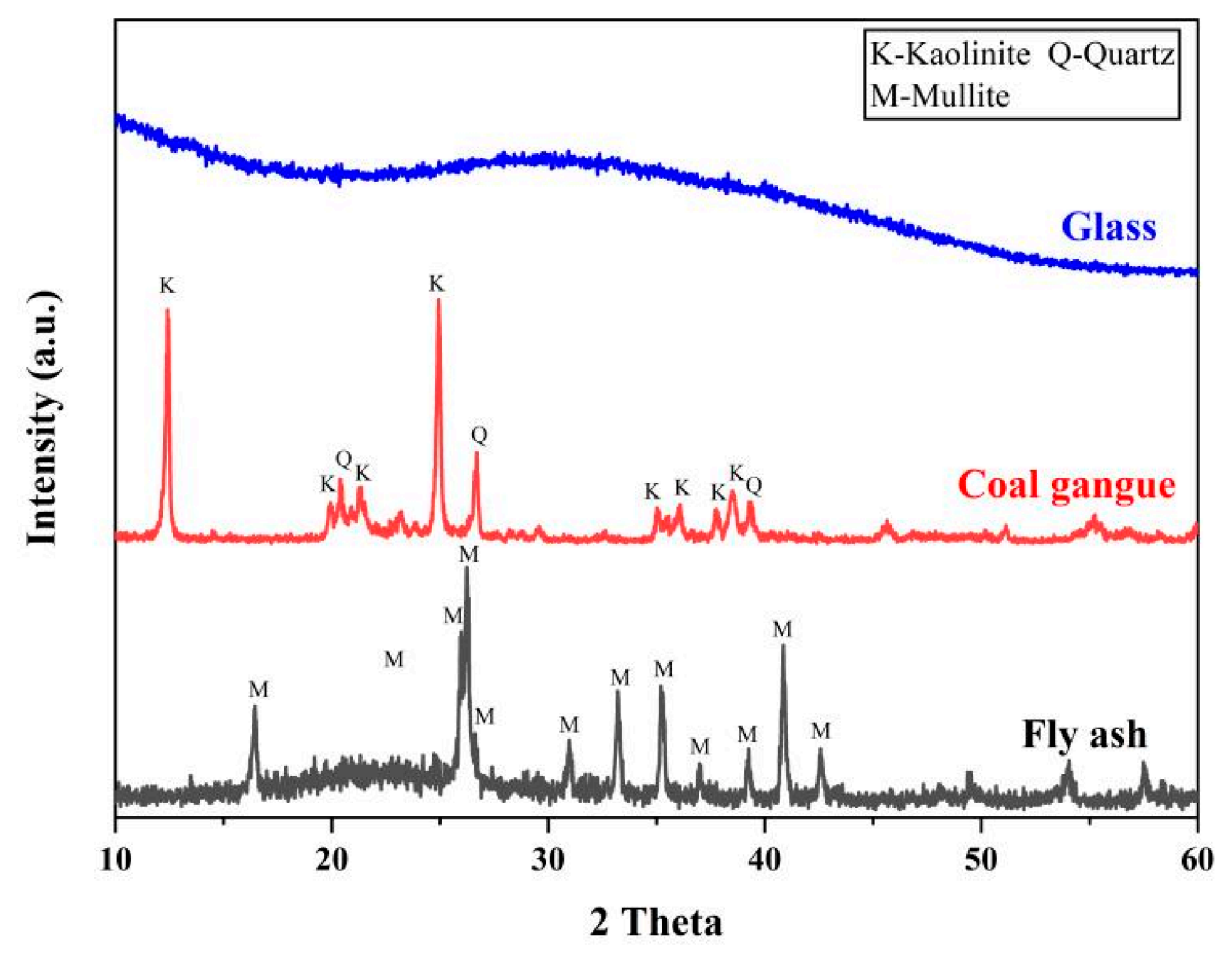
XRD patterns of the raw materials. M: mullite (PDF No. 01-079-1454); K: kaolinite (PDF No. 01-089-6538); Q: quartz (PDF No. 01-085-0795). The waste-glass pattern is predominantly amorphous.

The chemical compositions of the raw materials were determined by X-ray fluorescence (XRF, Zetium, PANalytical, Almelo, The Netherlands). Before analysis, each dried raw material was thoroughly homogenized and divided into three representative subsamples. XRF measurements were performed independently on the three subsamples, and the values reported in Table 1 are the arithmetic means of the triplicate determinations. The mineralogical phases were identified by X-ray diffraction (XRD, X-Pert3 Powder, PANalytical, The Netherlands) using the ICDD PDF database. As shown in Figure 1, the fly ash was dominated by mullite (PDF No. 01-079-1454) with a minor sillimanite phase (PDF No. 01-084-0983), together with a broad diffuse hump between 20° and 30° (2θ) arising from amorphous aluminosilicate material. The coal gangue mainly contained kaolinite (PDF No. 01-089-6538) and quartz (PDF No. 01-085-0795), whereas the waste glass was predominantly amorphous.

### 2.2. Formulation Design and Sample Preparation

**Figure 2:**
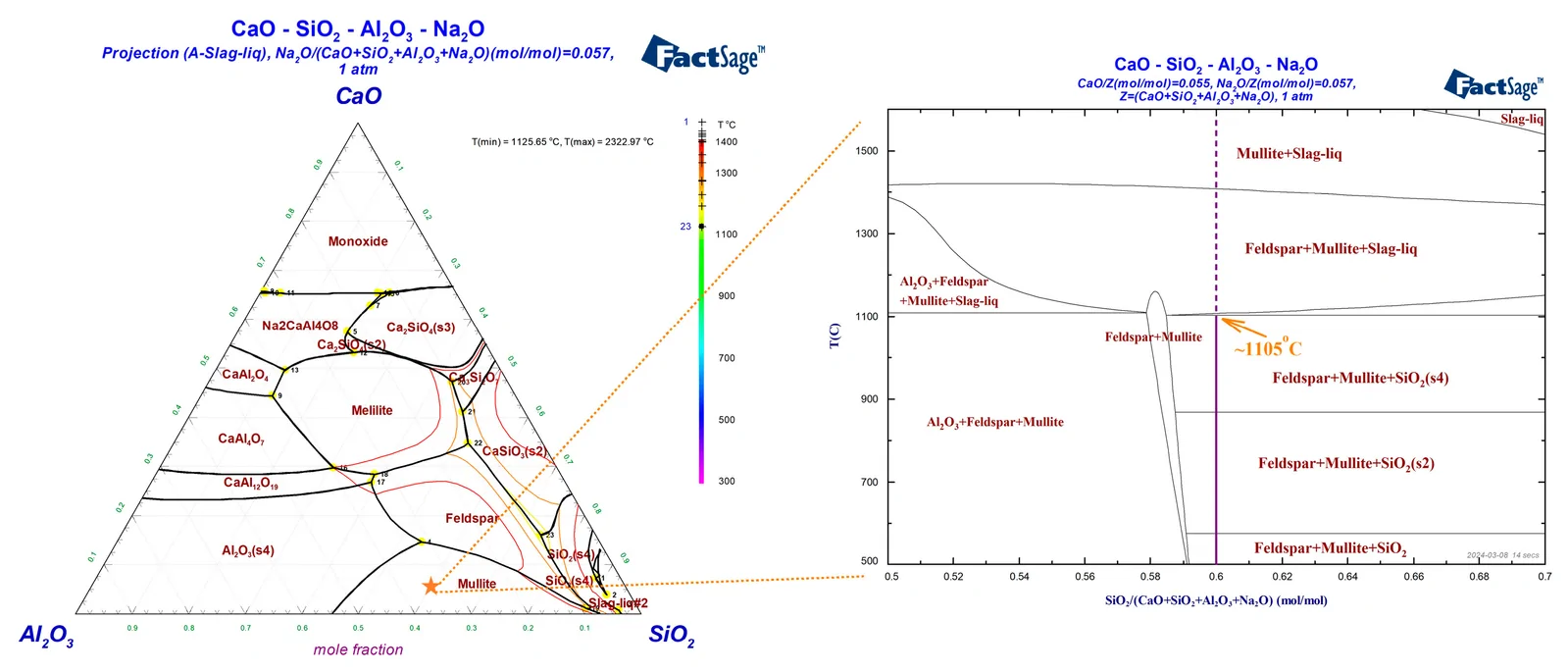
Phase-diagram-assisted composition analysis of the CaO-SiO_2_-Al_2_O_3_-Na_2_O system.

Based on the chemical characteristics of the raw materials and the compositional requirement for porous ceramsites, the formulation was designed with the aid of the CaO-SiO_2_-Al_2_O_3_-Na_2_O phase diagram, as shown in Figure 2. In aluminosilicate systems, the introduction of Na_2_O- and CaO-bearing fluxing components is favorable for reducing the liquid-phase formation temperature and promoting sintering. According to the phase-diagram-assisted composition analysis, a batch containing 60 mass % high-alumina fly ash, 10 mass % coal gangue, and 30 mass % waste glass was selected as the base formulation, and its calculated oxide composition is listed in Table 2.

The raw materials were homogenized in a planetary ball mill at 400 rpm for 12 h. After mixing, deionized water was added at 15 mass % of the dry raw-material mass, and the mixture was pelletized into spherical green bodies with a diameter of approximately 7–10 mm. The green pellets were dried at 105 °C for 3 h before sintering. Sintering was carried out in a muffle furnace. The samples were heated from room temperature to the target temperature at a heating rate of 10 °C/min, held for a designated duration, and then cooled naturally to room temperature in the furnace.

The experimental design is summarized in Table 3. For the temperature-dependent experiments, the sintering temperature was varied from 1200 to 1300 °C while the holding time was fixed at 120 min. For the holding-time experiments, the holding time was varied from 30 to 180 min at a fixed sintering temperature of 1280 °C.

### 2.3. Characterization, Property Testing, and Environmental Evaluation

The phase composition of the sintered products was analyzed by X-ray diffraction (XRD, X-Pert3 Powder, PANalytical, The Netherlands) using Cu Kα radiation at 40 kV and 40 mA over a 2θ range of 10–60° with a scanning rate of 4°/min. The microstructures of representative samples were observed by environmental scanning electron microscopy (ESEM, Quattro, Thermo Fisher, Waltham, MA, USA), and the local elemental compositions of selected phases were examined by energy-dispersive X-ray spectroscopy (ESEM-EDS, Quattro, Thermo Fisher Scientific, Waltham, MA, USA). The thermal behavior of the raw mixture was investigated by simultaneous thermogravimetric and differential scanning calorimetry (TG-DSC, Setaram S60/58341). Approximately 20 mg of sample was heated in an alumina crucible from room temperature to 1400 °C at a heating rate of 10 °C/min under an air atmosphere.

The bulk density of the sintered ceramsites was determined according to Archimedes’ principle using distilled water as the immersion medium. The mechanical performance was evaluated by measuring the compressive strength of cylindrical specimens (Φ30 × 30 mm) using a universal testing machine (Suns, Shenzhen, China) at a loading speed of 0.5 mm/min. Each measurement was conducted using 3 parallel samples, and the results are reported as the mean ± standard deviation.

For the determination of Ba, Cr, Mn, Pb, and Zn in the raw mixture and sintered samples, the solid samples were digested using a mixed HNO_3_/HF solution (9:1, *v*/*v*) and analyzed by ICP-AES (Prodigy 7, Leeman, Torrance, CA, USA). Certified reference materials GBW08401 and GBW07406 (GSS-6) were used for method calibration, and reagent blanks were analyzed under the same conditions. Two parallel determinations were performed for each sample, and the analytical precision was within ±10%. Environmental safety was evaluated using the Toxicity Characteristic Leaching Procedure (TCLP) in accordance with U.S. EPA Method 1311. A 0.1 mol/L acetic acid solution (pH 2.88) was used as the extraction fluid, and the liquid-to-solid ratio was 20:1 during a 24 h agitation period. After extraction, the leachates were filtered and analyzed by inductively coupled plasma atomic emission spectroscopy (ICP-AES, Prodigy 7, Leeman, USA). In addition, a long-term leaching test based on a modified NEN 7375 protocol was conducted in deionized water, 3.8 mass % NaCl solution, and simulated acid rain to evaluate the release behavior of impurity elements under freshwater, saline, and acidic environments. The leachates were collected at predetermined intervals of 1, 3, 7, 15, 30, and 46 days and analyzed by ICP-AES. “ND” indicates that the measured concentration was below the analytical detection limit.

## 3. Results and Discussion

### 3.1. Thermal Analysis of the Raw Mixture

**Figure 3:**
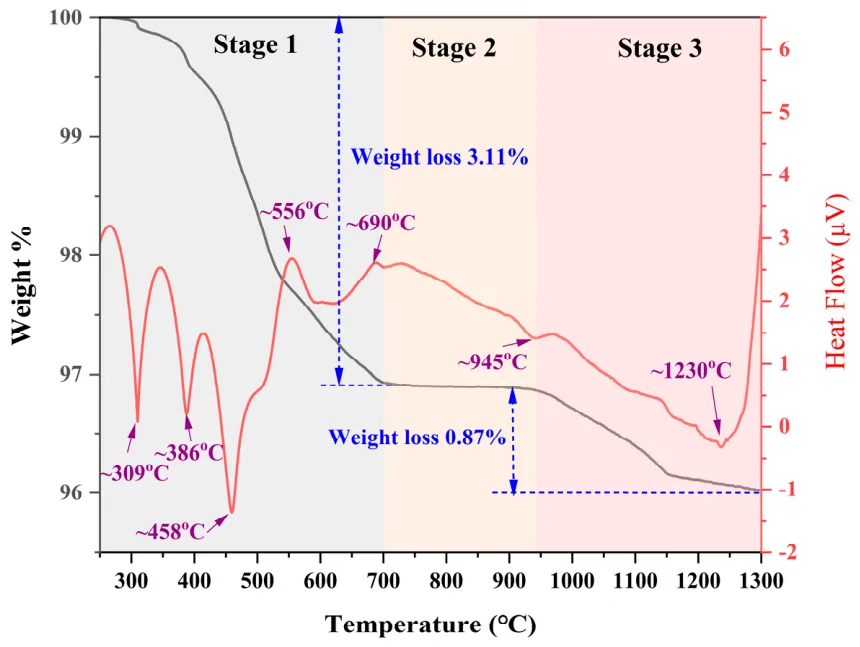
The TG-DSC curves of the raw mixture. The characteristic temperatures currently labeled in the figure denote peak temperatures (Tpeak).

Figure 3 presents the TG-DSC curves of the mixed raw materials. The thermal behavior indicates that the preparation of the ceramsites involved a series of coupled physicochemical processes, including dehydration and dehydroxylation, structural transformation of aluminosilicate phases, sulfate decomposition, and gradual liquid-phase formation. To distinguish reaction initiation from the peak extrema, the onset temperatures (Tonset) were estimated by the tangent-intersection method from the plotted DSC curve. The principal events showed Tonset values of approximately 287, 361, 426, 517, 637, 910, and 1143 °C, corresponding to Tpeak values of approximately 309, 386, 458, 556, 690, 945, and 1230 °C, respectively. These thermal events provide the thermochemical basis for the subsequent evolution of pore structure and mechanical properties during sintering.

Below approximately 700 °C, the main thermal events can be attributed to the removal of physically adsorbed water, the burnout of residual volatile matter from coal gangue, and the dehydroxylation of clay-like minerals. In this temperature range, the mass loss is mainly associated with unstable components in the raw mixture, while no obvious liquid-phase-assisted sintering is expected. Therefore, this stage mainly contributes to the initial mass reduction and structural activation of the raw materials before the onset of high-temperature reactions.

Between about 700 and 950 °C, the system undergoes further solid-state transformation and structural rearrangement. The weak thermal signal near 573 °C can be assigned to the quartz phase transition, whereas the broad endothermic behavior in this interval suggests the progressive transformation of aluminosilicate constituents. As the temperature approaches the upper end of this interval, the system gradually transitions toward sulfate decomposition and liquid-phase-assisted sintering. According to our previous work on high-alumina fly ash/waste-glass foam glass ceramics, pure CaSO_4_ decomposed at approximately 1220 °C in air, whereas its decomposition could be advanced to about 1020–1300 °C when incorporated into an aluminosilicate matrix, owing to interactions with SiO_2_- and Al_2_O_3_-bearing components [23]. Therefore, gas release from sulfate-bearing species is expected to occur near or above the upper limit of the present interval and to participate in pore formation at higher temperatures.

Above approximately 900 °C, the DSC curve becomes broader and the TG curve shows additional mass loss, indicating the onset of extensive liquid-phase formation accompanied by continued gas release. Because the raw mixture contains waste glass enriched in Na_2_O- and CaO-bearing components, the liquid phase is expected to form earlier and in larger quantity than in a glass-free aluminosilicate system. In this stage, eutectic reactions among silicate phases and fluxing oxides promote the formation of a viscous melt, while the gases released from sulfate decomposition and other thermally unstable components are retained within the melt, thereby favoring pore nucleation and growth. As the temperature further increases, however, excessive liquid-phase formation may reduce melt viscosity, accelerate pore coalescence, and weaken pore-wall integrity. Therefore, the final microstructure and mechanical performance of the ceramsites are governed by the balance among gas generation, liquid-phase development, and matrix consolidation. This interpretation is consistent with the subsequent evolution of bulk density, compressive strength, and pore morphology observed in the sintered samples.

Overall, the TG-DSC results confirm that the raw mixture possesses the thermal characteristics required for liquid-phase-assisted pore formation during firing. The key gas-evolving and melt-forming reactions occur before or within the selected sintering window, which supports the subsequent investigation of sintering temperature and holding time as the principal parameters governing the performance of the prepared ceramsites.

### 3.2. Effect of Sintering Temperature

**Figure 4:**
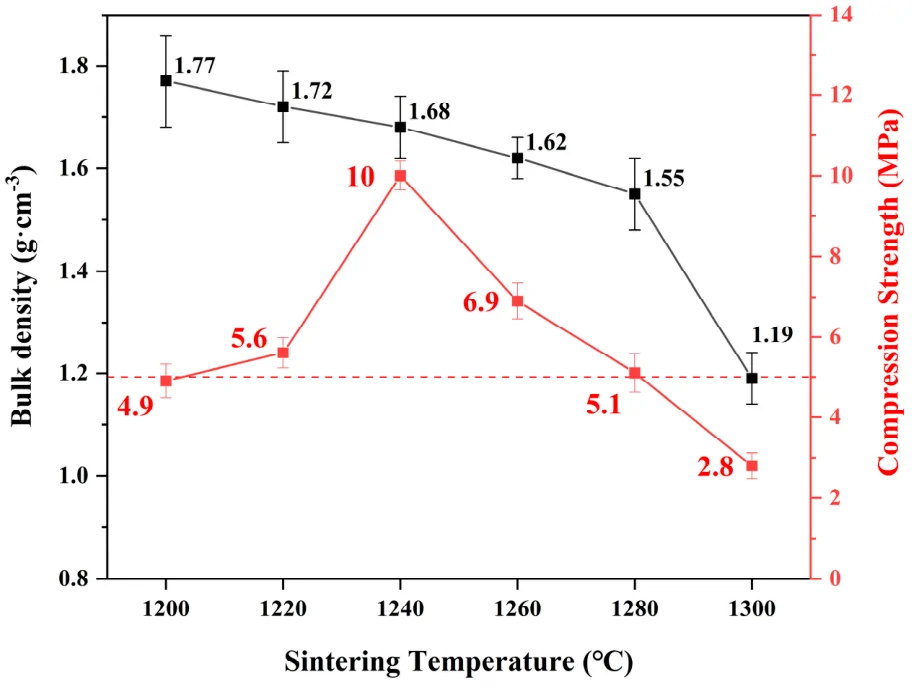
Bulk density and compressive strength of ceramsites sintered at different temperatures.

Figure 4 shows the effects of sintering temperature on the bulk density and compressive strength of the prepared ceramsites. As the sintering temperature increased, the bulk density decreased continuously, whereas the compressive strength first increased and then decreased. This trend indicates that sintering temperature plays a decisive role in regulating the balance between matrix consolidation and pore development in the present waste-derived ceramsite system. At relatively low temperatures, the amount of liquid phase is insufficient to promote effective interparticle bonding and structural densification. With increasing temperature, more liquid phase is generated, which enhances particle rearrangement and neck growth and thereby improves the load-bearing capacity of the ceramic skeleton. However, further temperature increase leads to excessive liquid-phase formation, which accelerates pore coalescence and pore-wall thinning, ultimately weakening the structural integrity of the ceramsites. The highest compressive strength, approximately 10 MPa, was obtained at 1240 °C. According to GB/T 17431-2010 [24], the cylinder compressive strength of ordinary lightweight aggregates should not be lower than 5 MPa. In the present study, samples sintered between 1220 and 1280 °C satisfied this requirement, whereas the specimens sintered at 1200 and 1300 °C did not. Therefore, 1240 °C can be regarded as the most favorable sintering temperature within the investigated range, because it provided the best compromise between pore development and mechanical stability.

**Figure 5:**
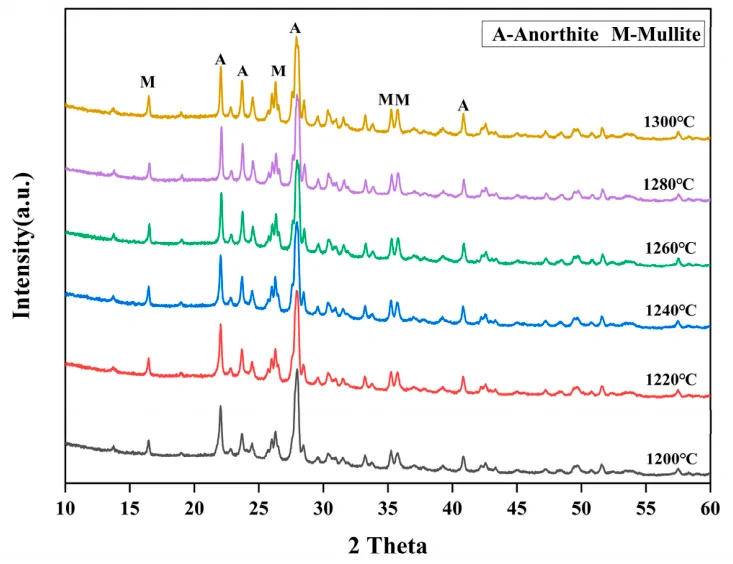
XRD patterns of ceramsites sintered at different temperatures. A: anorthite (PDF No. 00-041-1486); M: mullite (PDF No. 01-079-1454).

The XRD patterns shown in Figure 5 indicate that the phase assemblage of the ceramsites changed only slightly over the investigated temperature range and was dominated by anorthite (PDF No. 00-041-1486) and mullite (PDF No. 01-079-1454). No obvious quartz diffraction peak was identified in the sintered products. With increasing sintering temperature, the mullite diffraction peaks became slightly more pronounced, whereas the overall diffraction features remained broadly similar. This result suggests that the major phase composition was essentially stable and that temperature mainly affected the degree of crystallization rather than inducing pronounced phase transformation. The persistence of anorthite and mullite indicates that these two phases constituted the principal framework of the ceramic matrix. Therefore, the variations in bulk density and compressive strength with temperature were mainly governed by microstructural evolution, especially liquid-phase development and pore-structure adjustment, rather than by substantial differences in phase assemblage.

**Figure 6:**
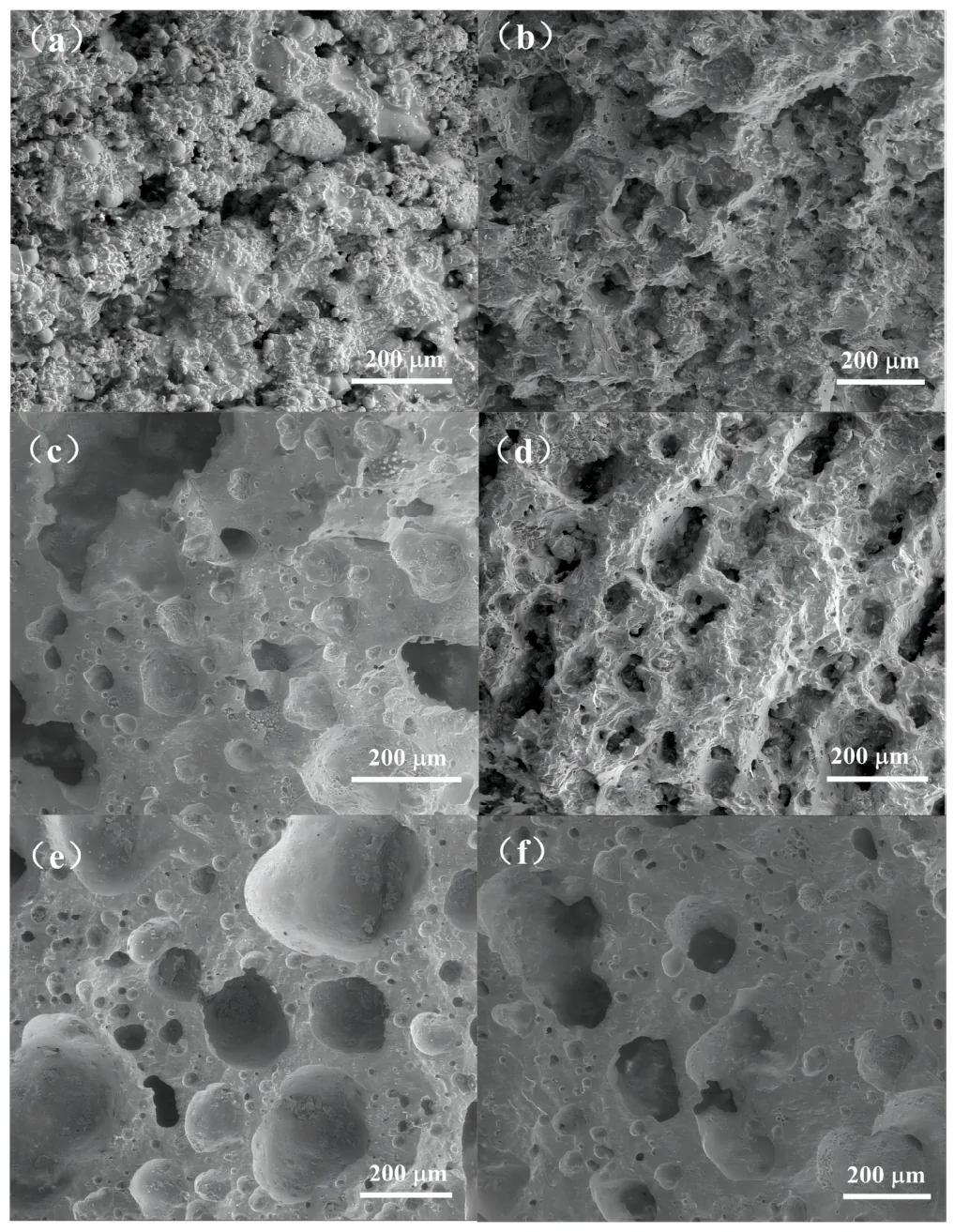
SEM micrographs of ceramsites sintered at different temperatures: (**a**) 1200 °C, (**b**) 1220 °C, (**c**) 1240 °C, (**d**) 1260 °C, (**e**) 1280 °C, and (**f**) 1300 °C.

The SEM images in Figure 6 show that the microstructure evolved significantly with increasing sintering temperature. At relatively low temperatures, the fracture surfaces were dominated by loosely bonded granular features, indicating insufficient liquid-phase formation and limited particle rearrangement. As the temperature increased, the surface became smoother and more continuous, suggesting that liquid phase was generated and spread between particles. Meanwhile, pores gradually appeared and developed, reflecting the combined effects of gas release and viscous-flow-assisted sintering. At 1240 °C, the ceramsite exhibited a relatively favorable balance between pore development and wall integrity, which corresponded to the highest compressive strength. When the temperature was further increased to 1260–1280 °C, pore development became more pronounced and the bulk density decreased continuously, but the pore walls also became thinner, leading to a decline in strength. At 1300 °C, the microstructure indicated over-sintering, characterized by excessive liquid-phase action, pore coalescence, gas escape, and local structural collapse. Although some pores appeared less distinct in the two-dimensional SEM view, this did not indicate beneficial densification; rather, it reflected the deterioration of skeletal continuity, which was consistent with the lowest bulk density and compressive strength observed at this temperature.

Combined with the thermal analysis discussed in Section 3.1, these results indicate that sintering temperature governs the final microstructure of the ceramsites by controlling the extent of liquid-phase formation and the interaction between gas release and viscous flow. Moderate liquid-phase formation favors particle bonding and the development of a relatively uniform porous structure, whereas excessive liquid formation promotes pore coarsening and reduces skeletal continuity. Therefore, the final properties of the ceramsites are controlled by the competition between densification and over-expansion, and 1240 °C represents the optimum temperature for achieving a favorable balance between low density and sufficient compressive strength in the present system.

### 3.3. Effect of Holding Time

**Figure 7:**
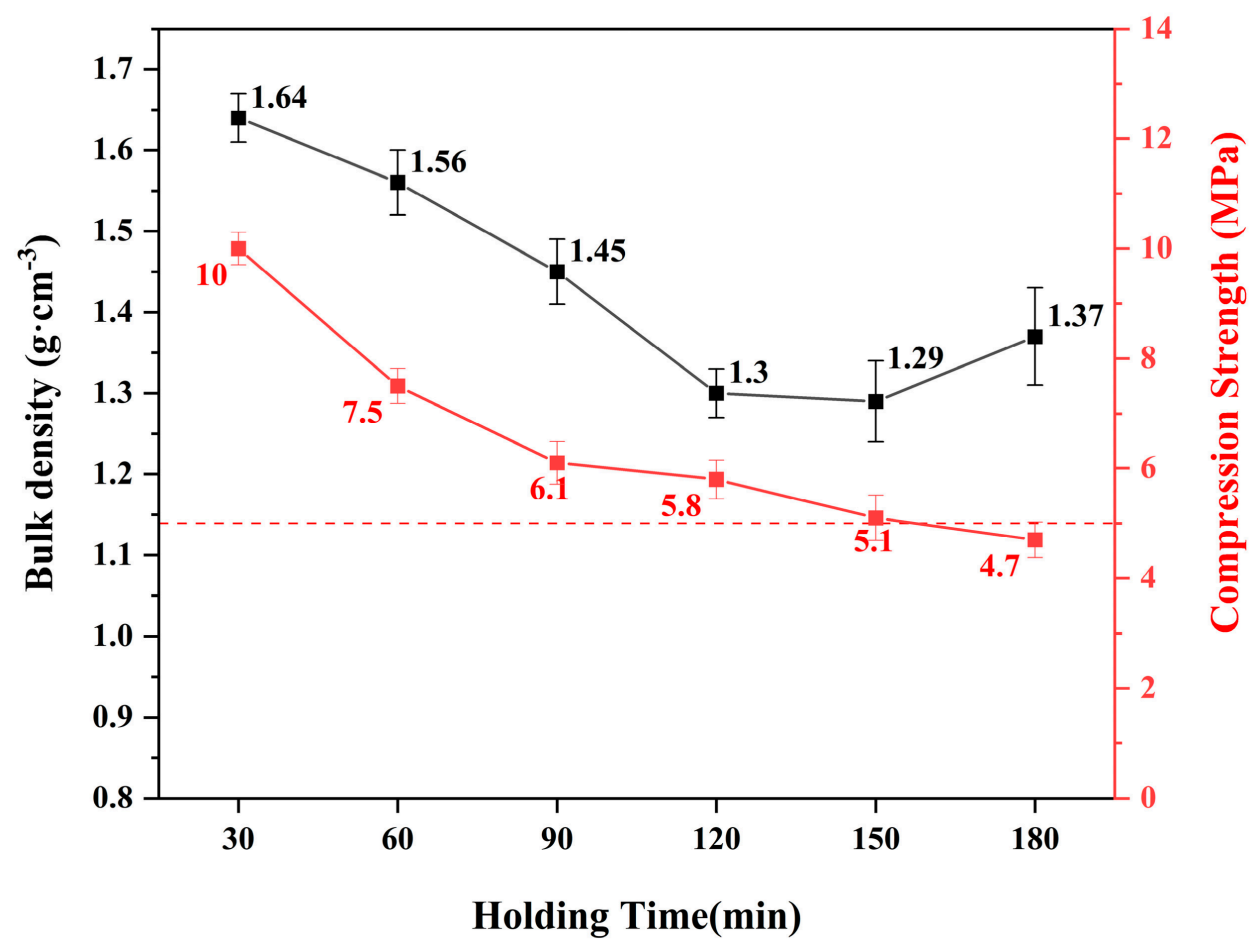
Bulk density and compressive strength of ceramsites sintered for different holding times at 1280 °C.

Figure 7 shows the effects of holding time on the bulk density and compressive strength of the ceramsites at a fixed sintering temperature of 1280 °C. As the holding time increased from 30 to 180 min, the bulk density first decreased from 1.64 to 1.29 g/cm^3^ and then slightly increased to 1.37 g/cm^3^, whereas the compressive strength continuously decreased from 10.0 to 4.7 MPa. These results indicate that holding time mainly affected the extent of pore coarsening and structural stability rather than promoting a substantial improvement in the load-bearing capacity of the ceramic skeleton. At a holding time of 30 min, the sample exhibited the highest bulk density and compressive strength, suggesting that the sintering process had already produced sufficient liquid phase to ensure effective particle bonding while maintaining relatively intact pore-wall structures. With prolonged holding, the compressive strength decreased progressively to 7.5 MPa at 60 min, 6.1 MPa at 90 min, 5.8 MPa at 120 min, and 5.1 MPa at 150 min. When the holding time was further extended to 180 min, the compressive strength declined to 4.7 MPa, which fell below the 5 MPa requirement specified in GB/T 17431-2010 for ordinary lightweight aggregates. Therefore, excessive holding time is unfavorable for maintaining the mechanical stability of the prepared ceramsites.

**Figure 8:**
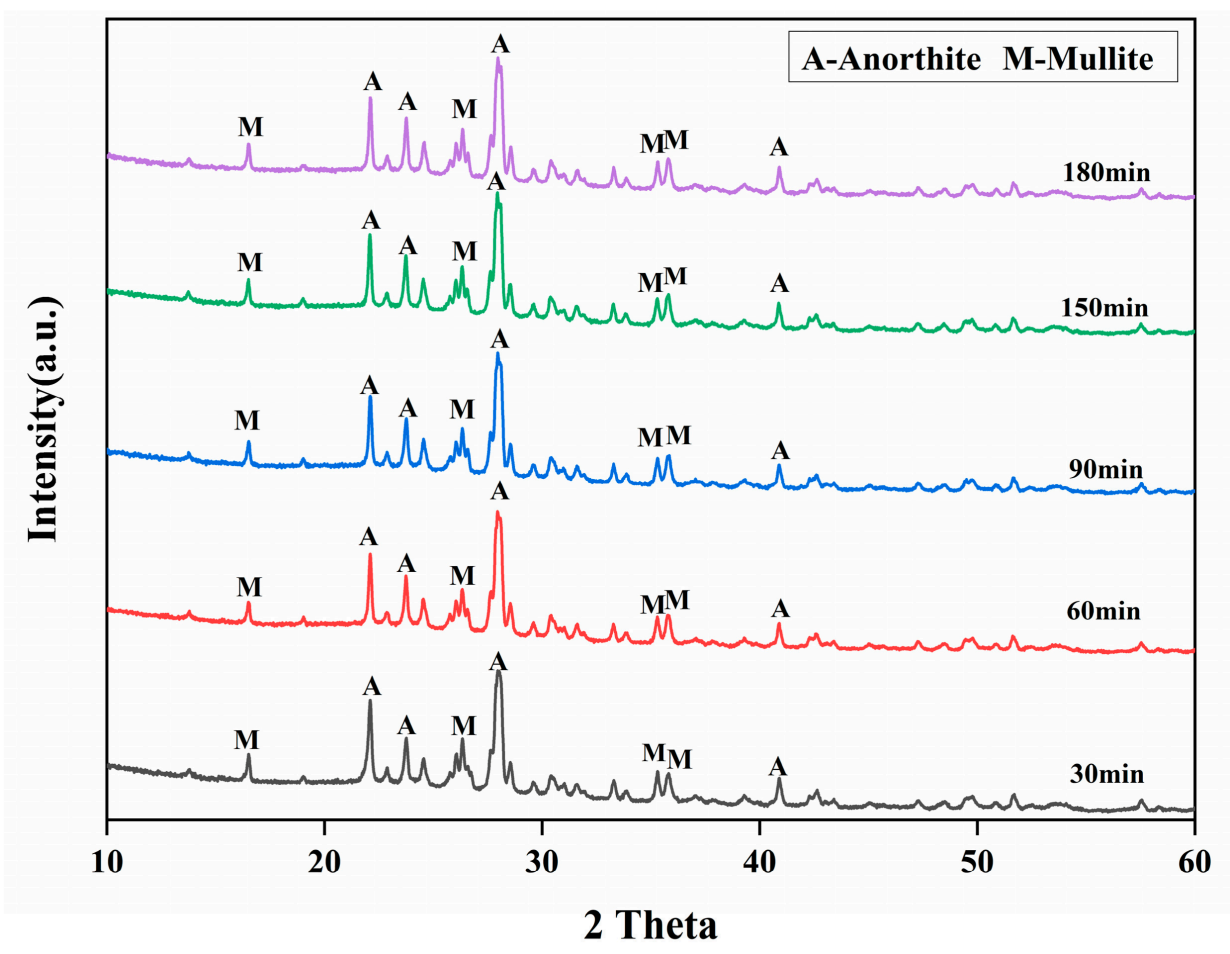
XRD patterns of ceramsites sintered for different holding times at 1280 °C. A: anorthite (PDF No. 00-041-1486); M: mullite (PDF No. 01-079-1454).

The XRD patterns shown in Figure 8 indicate that prolonging the holding time did not cause an obvious change in the crystalline phase assemblage. Anorthite (PDF No. 00-041-1486) and mullite (PDF No. 01-079-1454) remained the dominant crystalline phases throughout the investigated holding-time range, and no additional phase was identified reliably. The peak positions and relative intensities were broadly similar, indicating that at 1280 °C the principal phase-forming reactions had largely been completed and that further holding mainly affected pore coarsening and matrix rearrangement.

**Figure 9:**
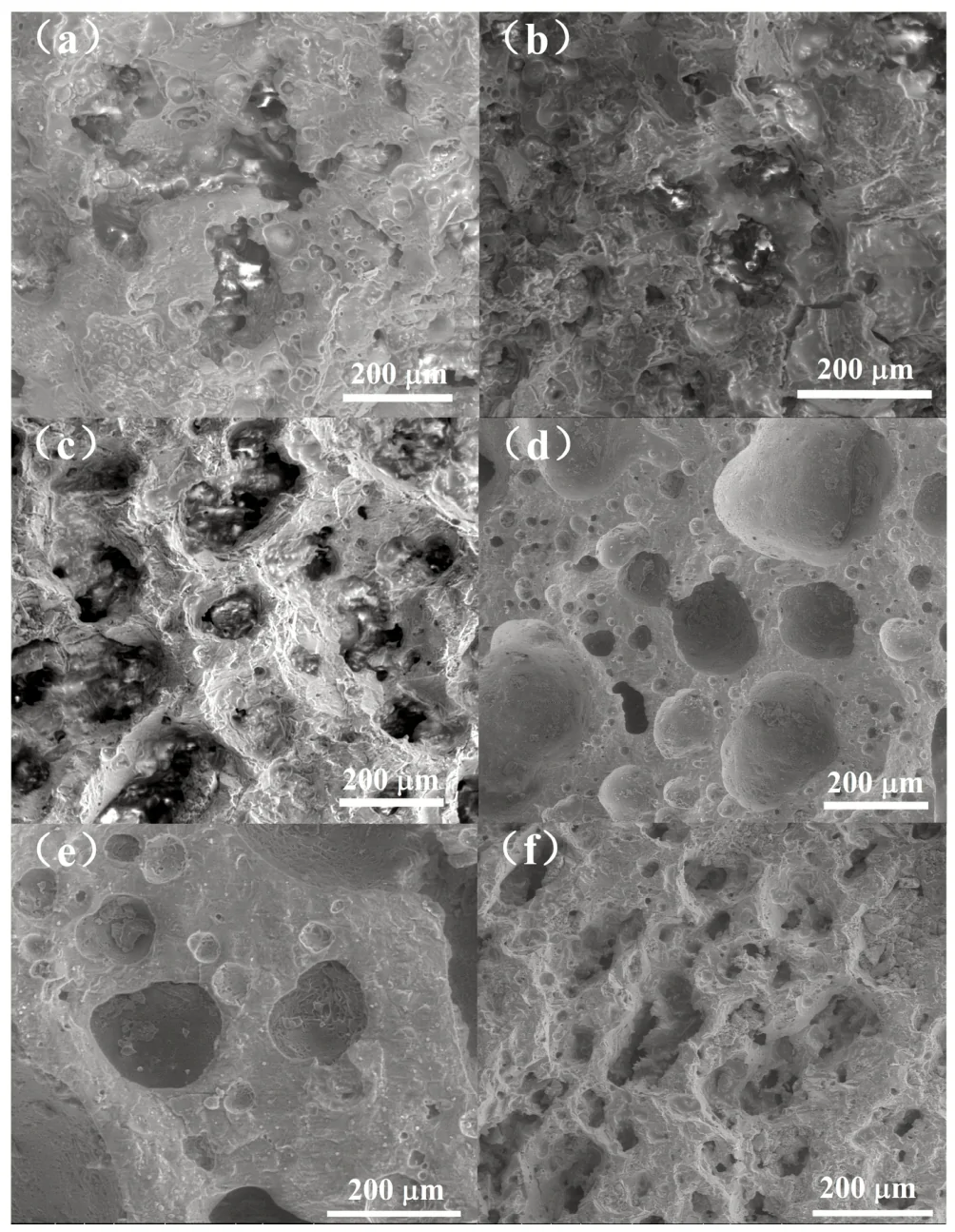
SEM micrographs of ceramsites sintered at 1280 °C for: (**a**) 30 min, (**b**) 60 min, (**c**) 90 min, (**d**) 120 min, (**e**) 150 min, and (**f**) 180 min.

The SEM images in Figure 9 further indicate that holding time mainly affected the evolution and stability of the pore structure. At short holding times, the pores were relatively small and incompletely developed, and the matrix still retained comparatively intact wall structures. With prolonged holding, more rounded and developed pores were formed, especially in the intermediate holding-time samples, indicating that continued liquid-phase action promoted gas diffusion and pore growth. However, this more developed porous morphology did not correspond to better mechanical performance, because the pore walls became progressively thinner and the continuity of the load-bearing skeleton was weakened. Therefore, although the intermediate holding-time samples exhibited a more obvious porous structure, their compressive strength was lower than that of the 30 min sample. When the holding time was extended further, the microstructure changed into a collapse-dominated porous morphology, in which pore coalescence, local shrinkage, and wall rupture occurred simultaneously. This was consistent with the slight rebound in bulk density and the further decrease in compressive strength at 180 min, indicating that excessive holding time led to structural coarsening rather than beneficial pore regulation.

Overall, holding time mainly regulated the coarsening and fusion behavior of pores rather than the major phase composition of the ceramsites. Its effect was therefore closely related to, but distinct from, that of sintering temperature. Short-to-moderate holding times were beneficial for preserving a more favorable balance between pore development and structural integrity, whereas excessive holding promoted pore coalescence, local collapse, and strength loss. Within the investigated range, 30 min was the most favorable holding time at 1280 °C for maintaining both low density and relatively high compressive strength.

### 3.4. Migration of Impurity Elements and Leaching Behavior

To evaluate the environmental safety of the waste-derived ceramsites, the migration behavior of selected impurity elements during sintering and their subsequent leaching characteristics were systematically investigated. As shown in Table 4, the apparent concentrations of Ba, Cr, Mn, and Zn in several sintered samples were higher than those in the unsintered raw mixture. For example, the Ba concentration reached 267.5 mg/kg in the sample sintered at 1260 °C, compared with 218.5 mg/kg in the raw mixture. In contrast, Pb decreased from 41 mg/kg at 1200 °C to 31 mg/kg at 1280 °C and then increased to 57 mg/kg at 1300 °C. These results indicate that the different impurity elements exhibited distinct apparent concentration changes during sintering.

The apparent increases in Ba, Cr, Mn, and Zn concentrations after sintering may result from the combined effects of mass loss from volatile components and retention or redistribution of these relatively less volatile elements within the liquid/glassy matrix. Therefore, an increase in concentration on a mass basis should not be interpreted alone as direct evidence of enhanced immobilization. Pb showed lower residual concentrations at 1200–1280 °C than in the raw mixture, suggesting partial volatilization during firing; however, the increase observed at 1300 °C indicates that redistribution and sample heterogeneity may also contribute. Without a complete elemental mass balance, the quantitative volatilization or retention fraction cannot be determined solely from the bulk concentration data. The environmental stability of the retained elements was therefore further evaluated using the subsequent TCLP and long-term leaching tests.

To further assess whether these retained elements posed an environmental risk, the leaching behavior of the ceramsites was evaluated by the Toxicity Characteristic Leaching Procedure (TCLP). As shown in Table 5, the TCLP leaching concentrations of all measured hazardous elements, including As, Ba, Cd, Cr, Pb, Hg, Se, and Ag, were far below the corresponding regulatory limits. For instance, the measured concentrations of Ba, Cr, Pb, and Se in the leachates were only 0.086, 0.016, 0.003, and 0.0011 mg/L, respectively, while several elements were not detected. These results demonstrate that, despite the retention of impurity elements in the sintered body, their mobility under acidic extraction conditions remained very low. Therefore, the high-temperature sintering process effectively reduced the short-term leaching risk of the prepared ceramsites.

The low TCLP leaching concentrations can be attributed to the combined effects of glassy encapsulation and structural stabilization within the ceramic matrix. During sintering, the waste-glass-derived liquid phase not only promoted densification and pore formation, but also provided a medium for immobilizing impurity elements. After cooling, these elements were retained in the solidified glassy phase and, at least partially, in relatively stable crystalline or interfacial structures. A similar interpretation has been adopted in recent Materials studies on waste-derived ceramsites, where heavy-metal stability was associated with incorporation into anorthite-bearing structures and encapsulation within dense glass phases [10].

To evaluate the long-term environmental compatibility of the prepared ceramsites under different service conditions, a multi-medium leaching test based on a modified NEN 7375 method was conducted in deionized water, 3.8 mass % NaCl solution, and simulated acid rain. As shown in Table 6, the long-term leaching concentrations of the monitored elements remained very low throughout the 46-day test period. Most elements, including Pb, Cr, Cu, and Co, were not detected in most cases, while the detected concentrations of Mn, Ni, Zn, and Ba remained at low levels. Although slight fluctuations were observed among different media and sampling times, no continuous increase or abnormal enrichment trend was found, indicating good long-term chemical stability of the sintered ceramsites under freshwater, saline, and acidic environments.

Among the three leaching media, the NaCl solution and simulated acid rain generally induced slightly higher release of some elements than deionized water, which is reasonable considering the higher ionic strength or acidity of these environments. Nevertheless, even under these relatively aggressive conditions, the measured concentrations remained very low, demonstrating that the ceramic matrix could effectively inhibit long-term migration of impurity elements. Therefore, the environmental safety of the prepared ceramsites was not only confirmed by short-term TCLP evaluation but also supported by their stable long-term performance in multiple simulated service environments.

Overall, the results indicate that the glass-assisted sintering process promoted the stabilization of impurity elements in the ceramsite matrix through liquid-phase retention and subsequent solidification. Although certain elements showed apparent enrichment in the sintered products, their actual leaching risk remained low. Consequently, the prepared ceramsites exhibited satisfactory short-term and long-term environmental safety, supporting their potential utilization as waste-derived ceramic aggregates.

## 4. Conclusions

Ceramsites with a high proportion of coal-based solid wastes were successfully prepared from high-alumina fly ash and coal gangue using waste glass as a fluxing additive. Based on the experimental results, the following conclusions can be drawn:

(1) Waste glass effectively promoted early liquid-phase formation in the CaO-SiO_2_-Al_2_O_3_-Na_2_O system and broadened the effective sintering window of the high-solid-waste formulation. As a result, the sintering regime strongly influenced pore evolution, matrix consolidation, and the final mechanical properties of the prepared ceramsites.
(2) Sintering temperature played a decisive role in determining the balance between pore development and structural integrity. As the temperature increased from 1200 to 1300 °C, the bulk density continuously decreased from 1.77 to 1.19 g/cm^3^, while the compressive strength first increased from 4.9 MPa to a maximum of 10.0 MPa at 1240 °C and then decreased to 2.8 MPa. Therefore, 1240 °C was identified as the optimum sintering temperature for achieving a favorable balance between low density and sufficient compressive strength. The main crystalline phases of the ceramsites were anorthite and mullite.
(3) Holding time mainly regulated pore coarsening rather than inducing substantial phase transformation. At 1280 °C, prolonging the holding time from 30 to 180 min caused the compressive strength to decrease continuously from 10.0 to 4.7 MPa, while the bulk density first decreased and then slightly increased. This behavior indicates that excessive holding promoted pore coalescence, pore-wall thinning, and local structural collapse, which weakened the load-bearing capacity of the ceramic matrix.
(4) The measured concentration changes in impurity elements during sintering were consistent with the combined effects of mass loss, partial volatilization, and retention or redistribution within the liquid/glassy matrix. Although Ba, Cr, Mn, and Zn showed apparent concentration increases in some sintered samples, their actual leaching risk remained low. TCLP results showed that all measured hazardous-element concentrations were far below the corresponding regulatory limits, and the long-term leaching test further confirmed very low release levels under freshwater, saline, and acidic environments.
(5) Overall, glass-assisted sintering provides an effective route for converting coal-based solid wastes into environmentally safe ceramsites. The results clarify the processing–structure–property relationship of high-alumina fly ash–coal gangue ceramsites and demonstrate their potential as sustainable waste-derived lightweight ceramic aggregates.

## Figures and Tables

**Table 1:** Chemical composition of the raw materials (mass %).

Raw Materials	SiO_2_	Al_2_O_3_	CaO	MgO	Fe_2_O_3_	Na_2_O	K_2_O	LOI
Fly ash	46.93	43.40	3.16	0.35	2.18	0.09	0.41	1.54
Coal gangue	39.62	30.04	2.22	0.29	0.79	0.00	0.34	26.7
Waste Glass	66.55	1.55	8.71	4.55	0.26	17.26	0.55	-

Note: Each value is the mean of three independent XRF measurements performed on homogenized representative subsamples.

**Table 2:** Calculated oxide composition of the selected batch (mass %).

Mass %	SiO_2_	Al_2_O_3_	CaO	MgO	Fe_2_O_3_	Na_2_O	K_2_O	LOI
Sample	52.08	29.51	4.73	1.60	1.47	5.23	0.44	3.59

**Table 3:** Experiment Scheme and Design.

Sample	Sintering Temperature (°C)	Holding Time (min)
S-1	1200	120
S-2	1220	
S-3	1240	
S-4	1260	
S-5	1280	
S-6	1300	
S-7	1280	30
S-8		60
S-9		90
S-10		150
S-11		180

**Table 4:** Contents of selected elements in the raw mixture and samples sintered at different temperatures (mg/kg).

Elements	Raw Materials	S-1	S-2	S-3	S-4	S-5	S-6
Ba	218.5	216.5	228	228.5	267.5	224	226.5
Cr	75	96	90.5	85.5	76	82.5	77.5
Mn	106	110	114.5	112.5	128.5	109	115
Pb	63	41	39	32	30.5	31	57
Zn	86	94	95.5	90.5	87	88	100

Note: Values were obtained from duplicate ICP-AES determinations; the analytical precision was within ±10%.

**Table 5:** TCLP leaching concentrations of selected hazardous elements in the ceramsites (mg/L).

Heavy Metals	As	Ba	Cd	Cr	Pb	Hg	Se	Ag
Leachates of ceramsites	ND	0.086	ND	0.016	0.003	ND	0.0011	ND
Limiting value	5	100	1	5	5	0.2	1	5

ND indicates a concentration below the analytical detection limit. Regulatory limits are from 40 CFR 261.24.

**Table 6:** Long-term leaching concentrations of selected elements from the ceramsites under different simulated service environments (mg/L).

Medium	Element	1	3	7	15	30	46
Deionized water	Ba	ND	ND	ND	ND	ND	ND
	Cd	ND	ND	ND	ND	ND	ND
	Cr	ND	ND	ND	ND	ND	ND
	Cu	ND	ND	ND	ND	ND	ND
	Mn	0.0005	0.0006	0.0007	0.0008	0.0008	0.0008
	Ni	0.0028	0.0059	0.0093	0.0087	0.0073	0.0068
	Pb	ND	ND	ND	ND	ND	ND
	Zn	ND	0.0981	ND	0.0471	0.045	ND
	Co	ND	ND	ND	ND	ND	ND
NaCl solution	Ba	0.0952	0.1135	0.1001	0.0779	0.034	0.0401
	Cd	ND	ND	ND	ND	0.0003	0.0001
	Cr	ND	ND	ND	ND	ND	ND
	Cu	ND	ND	ND	ND	ND	ND
	Mn	0.0015	0.0013	0.0012	0.0008	0.0005	0.0006
	Ni	ND	0.0052	0.0069	0.0036	0.0057	0.0038
	Pb	ND	ND	ND	ND	ND	ND
	Zn	ND	0.0836	0.011	0.0168	0.007	ND
	Co	ND	ND	ND	ND	ND	ND
Simulated acid rain	Ba	0.0368	0.0198	0.0128	0.012	0.0046	0.0006
	Cd	0.0018	0.0004	ND	ND	ND	ND
	Cr	ND	ND	ND	ND	ND	ND
	Cu	ND	ND	ND	ND	ND	ND
	Mn	0.0003	0.0005	0.0006	0.0002	0.0006	0.0004
	Ni	0.0011	0.0053	0.0112	0.0085	0.0067	0.0066
	Pb	ND	ND	ND	ND	ND	ND
	Zn	ND	0.0378	0.0116	0.0291	0.0219	ND
	Co	ND	ND	ND	ND	ND	ND

ND indicates concentrations below the detection limit or negative values after blank/background correction.

## Data Availability

The original contributions presented in this study are included in the article. Further inquiries can be directed to the corresponding author.
